# Conversion of Glucose to 5-Hydroxymethylfurfural in a Microreactor

**DOI:** 10.3389/fchem.2019.00951

**Published:** 2020-01-22

**Authors:** Tiprawee Tongtummachat, Nattee Akkarawatkhoosith, Amaraporn Kaewchada, Attasak Jaree

**Affiliations:** ^1^Department of Chemical Engineering, Faculty of Engineering, Mahidol University, Nakhon Pathom, Thailand; ^2^Department of Agro-Industrial, Food and Environmental Technology, King Mongkut's University of Technology North Bangkok, Bangkok, Thailand; ^3^Department of Chemical Engineering, Faculty of Engineering, Center of Excellence on Petrochemical and Materials Technology, Kasetsart University, Bangkok, Thailand

**Keywords:** glucose, 5-hydroxymethylfurfural, microreactor, homogeneous catalyst, dispersed-flow

## Abstract

5-hydroxymethylfurfural (5-HMF) is one of the key bio-based platform chemicals for the production of high-value chemicals and fuels. The conventional production of 5-HMF from biomass is confronted by the relatively low yield and high production cost. In this work, the enhancement of a continuous catalytic synthesis of 5-HMF in a biphasic-dispersed flow reactor was proposed. Glucose, hydrochloric acid, and methyl isobutyl ketone (MIBK) were used as a low-cost raw material, catalyst, and organic solvent, respectively. The main factors (reaction temperature, residence time, solvent amount, and catalyst concentration) affecting the yield and selectivity of 5-HMF were studied. The 5-HMF yield of 81.7% and 5-HMF selectivity of 89.8% were achieved at the residence time of 3 min, reaction temperature of 180°C, the volumetric flow rate of aqueous phase to organic phase of 0.5:1, and catalyst concentration of 0.15 M. The yield and selectivity of 5-HMF obtained from the biphasic system were significantly higher than that obtained from the single phase system. The superior 5-HMF production in our system in terms of operating conditions was presented when compared to the literature data. Furthermore, the continuous process for removing HCl from the aqueous product was also proposed.

## Introduction

Nowadays, the manufacturing of fuels and chemicals from non-renewable sources has raised growing environmental concerns. This led to research and development promoting the use alternative sources of energy instead of conventional fossil-based sources. One of the promising strategies is to transform biomass into high-value chemical substances. Among the biomass-based materials, 5-Hydroxymethylfurfural (5-HMF) nowadays has received a lot of attention due to the possibility of converting it into various high-value products such as 5-furandicarboxylic acid, 2,5-dimethyltetrahydrofuran, and 2,5-bis(hydroxymethyl)furan (Rosatella et al., 2011).

5-HMF is generally produced through the dehydration reaction of fructose with a presence of catalyst in order to achieve high conversion and selectivity; however, this technique is restricted by the inherent disadvantages posed by the high production cost and limited accessibility of fructose (Agarwal et al., 2018). Recently, many researchers have attempted to find effective substrates for producing 5-HMF such as glucose because of the low production cost and uninterrupted supply (Hu et al., 2013). Unfortunately, using glucose as a substrate to produce 5-HMF normally involves the isomerization of glucose to fructose and dehydration of fructose to 5-HMF, leading to the catalyst requirement of both Lewis acid and Brønsted acid for isomerization and dehydration, respectively. The relatively low yield and selectivity are the consequence of using this substrate (Rosatella et al., 2011). To overcome the yield issue, many different routes and methods have been proposed. For instance, supercritical conditions was reported to enhance the synthesis of 5-HMF from glucose (Watanabe et al., 2005b). However, the equipment and operating cost are still the major issues for this technique. As an alternative solution, many novel heterogeneous catalysts have been developed (Yang et al., 2015; Xin et al., 2017). Yet, this method requires a complicated catalyst preparation procedure. The synthesis of 5-HMF via the direct dehydration of glucose can be possible with the use of a very high concentration of Brønsted acid (Li et al., 2017). Unfortunately, the slow rate of reaction is one of the major obstacles for this route.

Microreactor technology has emerged as one of the attractive solutions for many applications confronted by the transport phenomena issues (Yao et al., 2015). High productivity with low production costs is another highlight of this technology because it involves a continuous production process. For the production of 5-HMF from glucose, microreactor technology was recently introduced to enhance the yield and selectivity of 5-HMF as reported by Guo et al. (2019) using bifunctional homogeneous catalysts (Lewis and Brønsted acids). High-throughput 5-HMF was successfully achieved through this technique due to efficient extraction of 5-HMF from the aqueous phase compared to the conventional reactors. A slug flow pattern was also observed from their works providing small liquid-liquid interfacial areas for mass transfer compared to the dispersed-flow condition which would conceivably require a shorter contact time (Dore et al., 2012).

Based on the literature, 5-HMF production via the direct dehydration of glucose under dispersed flow conditions has never been investigated. Therefore, in this work, we proposed a simple and effective 5-HMF synthesis through the dehydration of glucose using commercial hydrochloric acid as catalyst by applying the microreactor technology. The effect of operating conditions, including reaction temperature, residence time, catalyst concentration, and organic-to-aqueous volumetric ratio on the 5-HMF yield and selectivity was investigated. The performance of 5-HMF synthesis in both monophasic and biphasic systems was evaluated. The production performance of our method was compared to that of other production techniques. For purification of product, we also developed a continuous method for the removal of HCl from aqueous product.

## Materials and Methods

### Materials

Glucose and fructose (AR grade) were purchased from Fisher Scientific. 37% (w/w) hydrochloric acid (HCl) and methyl isobutyl ketone (MIBK) were obtained from Merck. Analytical standard of 5-HMF was purchased from Sigma-Aldrich. Acetonitrile (HPLC grade) was acquired from Honeywall and was used as a mobile phase for product analyst.

### 5-HMF Synthesis

**Figure 1:**
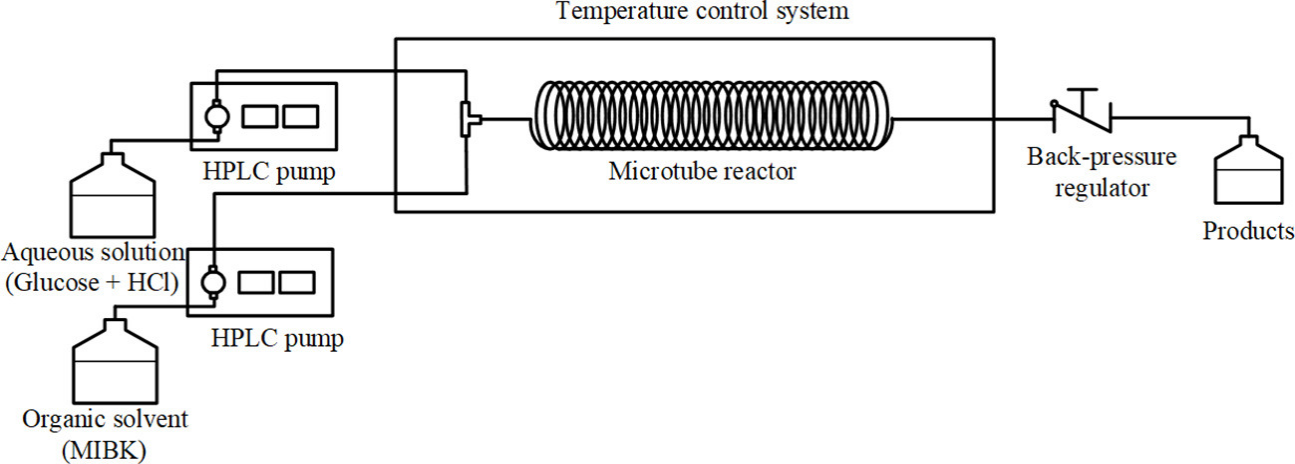
Schematic diagram of the experimental apparatus.

Prior to the experiment, glucose, deionized (DI) water, and HCl were uniformly mixed in a beaker at various HCl concentration levels, whereas the glucose concentration was kept constant at 5 g/L for all experiments. MIBK was used as an organic solvent for extraction of 5-HMF during the synthesis. The aqueous phase (glucose and HCl dissolved in water) and the organic phase (MIBK) were separately fed into the T-micromixer via HPLC pumps at different flow rates depending on the residence time and the ratio of organic to aqueous phase investigated. Before entering the micromixer, both liquid streams were preheated to the desired temperature. After mixing, the two-phase mixture entered the microtube reactor (ID: 0.87 mm, 4 mL) where the reactions took place. The micromixer and microreactor were placed in a convection oven to control the reaction temperature. To avoid the vaporization of solvent, a back-pressure regulator was installed at the exit end of the reactor to control the pressure of the system at 80 bar. The outlet stream from the back-pressure regulator was quenched to stop any further reaction. The product, in both organic and aqueous phases, was then collected in order to evaluate the amount of 5-HMF as well as the reaction performance, including conversion, selectivity, and yield. Figure 1 represents the experimental setup of this work.

### Product Analysis

The amount of 5-HMF was determined by HPLC technique with UV detector (UV; model 2550, Varian) at 320 nm. The ACE C_18_ column (4.6 mm × 250 mm, 5 μm particle size, Advanced Chromatography Technologies) was applied as a separation column, which was operated at the temperature of 35°C. The mobile phase solution of water and acetonitrile [90:10 (V/V)] was used at a flow rate of 0.7 mL/min. The identification of peaks was based on the comparison of the retention time between the sample and that of the standard compounds. The external standard was used to quantitate the amount of 5-HMF. The side reaction products including formic acid and levulinic acid were detected under the similar conditions as the analysis of HMF except that the UV wavelength was 295 nm.

The remaining monosaccharides (glucose and fructose) was analyzed by HPLC equipped with RI detector (RI; model YL9170, YL Instrument). The analysis was performed on the APS-2 Hypersil (4.6 mm × 250 mm, 5 μm particle size, Thermo Scientific) at the temperature of 35°C. A stream of 75:25 (v/v) acetonitrile: water was applied as a mobile phase at the flow rate of 0.7 mL/min.

The production performance of our method was designated by several parameters as described below.

(1) $$\begin{array}{l} Glucose\;conversion\left(\%\right)\\ \begin{array}{c} =\left(1-\left(\frac{Moles\;of\;glucose\;unreacted}{Moles\;of\;starting\;glucose}\right)\right)\times 100 \end{array} \end{array}$$

(2) $$\begin{array}{r} 5-HMF\;Yield\left(\%\right)=\left(\frac{Moles\;of\;HMF\;produced}{Moles\;of\;starting\;glucose}\right)\times 100 \end{array}$$

(3) $$\begin{array}{r} 5-HMF\;selectivity\left(\%\right)=\left(\frac{5-HMF\;yield}{Glucose\;conversion}\right)\times 100 \end{array}$$

(4) $$\begin{array}{l} 5-HMF\;extraction\;efficiency\left(\%\right)\\ \begin{array}{c} =\left(\frac{Moles\;of\;HMF\;existed\;in\;organic\;phase}{Moles\;of\;HMF\;produced}\right)\times 100 \end{array} \end{array}$$

## Results and Discussion

### 5-HMF Synthesis in a Monophasic System

**Figure 2:**
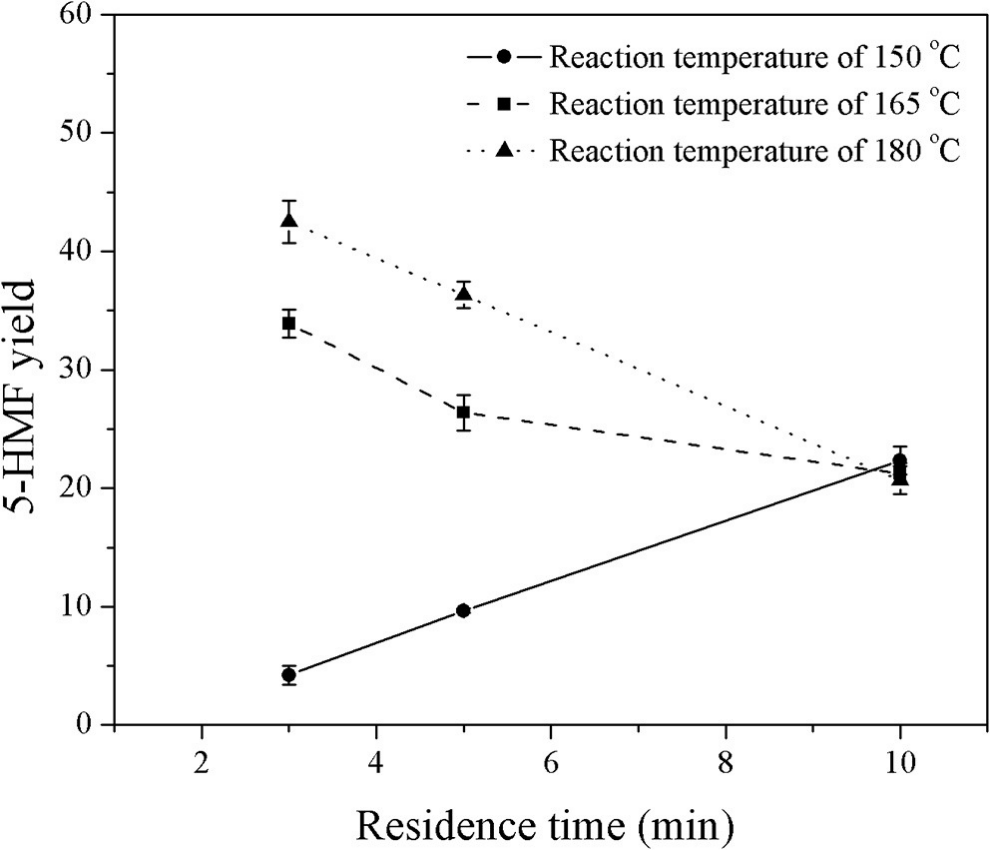
Effect of reaction temperature on the yield of 5-HMF produced in a monophasic system at various residence times.

**Figure 3:**
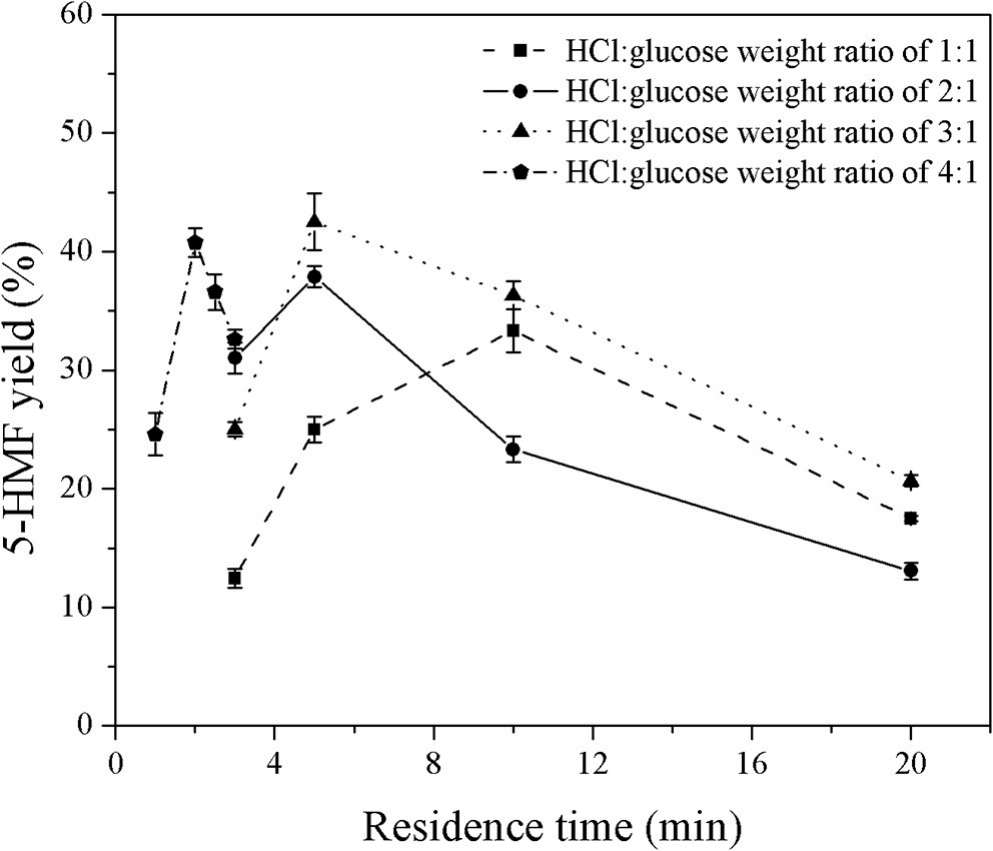
Effect of catalyst concentration on the yield of 5-HMF produced in a monophasic system under various residence times.

First, the monophasic system was used to study the behavior of 5-HMF in a microreactor system. Note that the monophasic synthesis of 5-HMF from glucose in a microreactor has hardly been investigated. The experiments were performed under various reaction temperatures (150–180°C), residence times (3–10 min), and HCl-to-glucose weight ratios (1:1 to 4:1). For the HCl-to-glucose weight ratio, the weight of HCl and glucose were based on the 37% (w/w) HCl and the weight of anhydrous glucose powder, respectively. The results are shown in Figures 2, 3.

Figure 2 shows the effect of reaction temperature on the yield of 5-HMF at a constant HCl-to-glucose weight ratio of 3:1, while the residence time was varied in the range of 3–10 min. The results show that the yield of 5-HMF increased with increasing reaction temperature. For example, at a residence time of 5 min, the 5-HMF yield increased from 9.7 to 36.3% with an increase in reaction temperature from 150 to 180°C, since the dehydration of glucose to 5-HMF is highly endothermic reaction (Assary et al., 2010). This means that an increase in the reaction temperature favors the dehydration reaction that converts glucose into 5-HMF. Note that a further increase in reaction temperature of more than 200°C might cause the decomposition of glucose to undesired products such as anhydroglucose and glyceraldehyde (Matsumura et al., 2006; Souza et al., 2012). The highest yield of 42.5% was achieved at a reaction temperature of 180°C. Therefore, this reaction temperature was selected for further experiments of 5-HMF synthesis in this work, which was in line with many related reports (Zhang et al., 2015; Muranaka et al., 2017). Additionally, at the reaction temperature of 165 and 180°C, the 5-HMF yield decreased with increasing residence time. This was because 5-HMF produced rapidly at the early stage of the reaction was subsequently converted into formic and levulinic acids through the hydration reaction. On the other hand, at the reaction temperature of 150°C, the yield of 5-HMF increased almost linearly as the residence time increased. At this temperature, the system was not greatly affected by the rehydration of 5-HMF because the production rate of 5-HMF was relatively slow.

**Figure 4:**
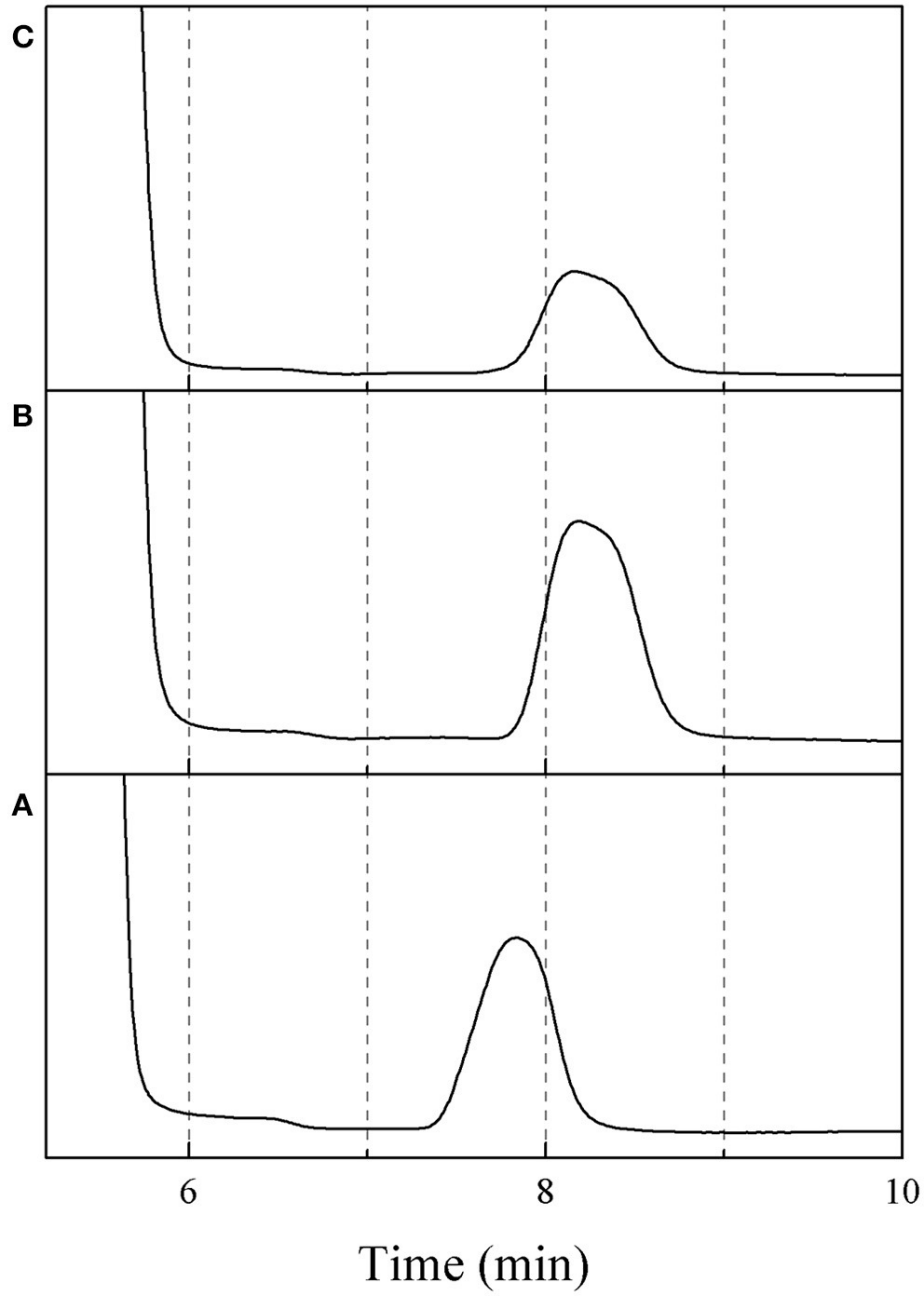
HPLC chromatograms of **(A)** fructose, **(B)** glucose, **(C)** sample (reaction condition: HCl-glucose ratio of 1:1, residence time of 5 min and reaction temperature of 180°C).

The effect of residence time on the yield of 5-HMF was further investigated under various HCl-to-glucose weight ratios (represented as a catalyst concentration), as shown in Figure 3. To study this effect, the reaction temperature was kept constant at 180°C. For the HCl-to-glucose weight ratio of 1:1, the 5-HMF yield increased from 12.4 to 25.0% when the residence time was changed from 3 to 5 min and then reached the maximum value of 33.3% at the residence time of 10 min. However, the prolonged residence time exceeding 10 min provided a negative impact on the yield of 5-HMF due to the transformation of the 5-HMF into other by-products. This was similar to results obtained using the HCl-to-glucose weight ratio of 2:1, 3:1, and 4:1. Besides, a trace of glucose was detected in the product outlet, as shown in the HPLC chromatogram (see Figure 4). This implies that the direct dehydration of glucose to 5-HMF occurred in the system when only the Brønsted acid was applied for 5-HMF synthesis.

Moreover, an increase in catalyst concentration significantly shortened the residence time required to achieve the maximum yield (see Figure 3). For example, it took 10 min to reach the maximum yield of 33.3% when using the weight ratio of HCl to glucose of 1:1, while the residence time of only 5 min was sufficient to achieve the maximum yield of 37.8% for the weight ratio of HCl to glucose of 2:1. From this result, at a reaction temperature of 180°C, the highest yield of 42.5% was achieved at the weight ratio of HCl to glucose of 3:1 and residence time of 3 min. It was apparent that our monophasic synthesis in a microreactor provided superior performance compared to the conventional stirred batch reactor carrying out the monophasic synthesis of 5-HMF. For instance, the yield of 5-HMF below 10% was obtained from the conversion of glucose using mineral acid as catalyst operating for the reaction temperature and residence time in the range of 170–200°C and 3–20 min, respectively (Mednick, 1962; Watanabe et al., 2005a; Qi et al., 2008). Nevertheless, the yield of 5-HMF in our system was still too low; therefore, the development of our technique was further extended to the application of a biphasic system.

### 5-HMF Synthesis in a Biphasic System

**Figure 5:**
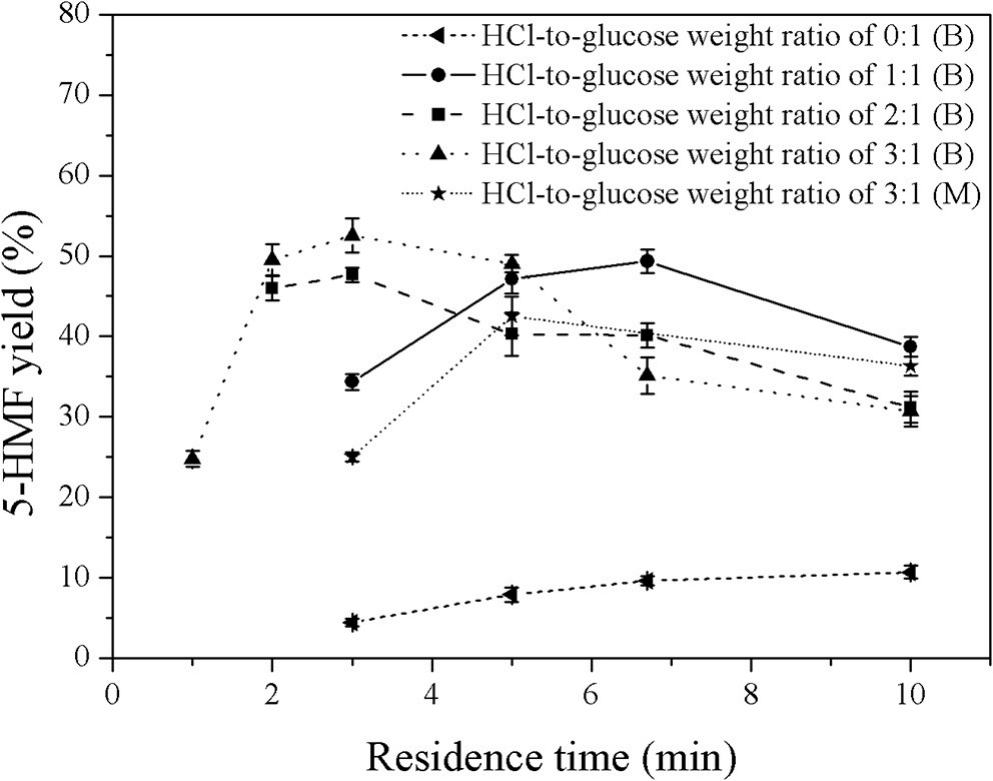
Effect of catalyst concentration on the 5-HMF yield in a biphasic system under various residence times. (B) and (M) represent the biphasic system and monophasic system, respectively.

**Figure 6:**
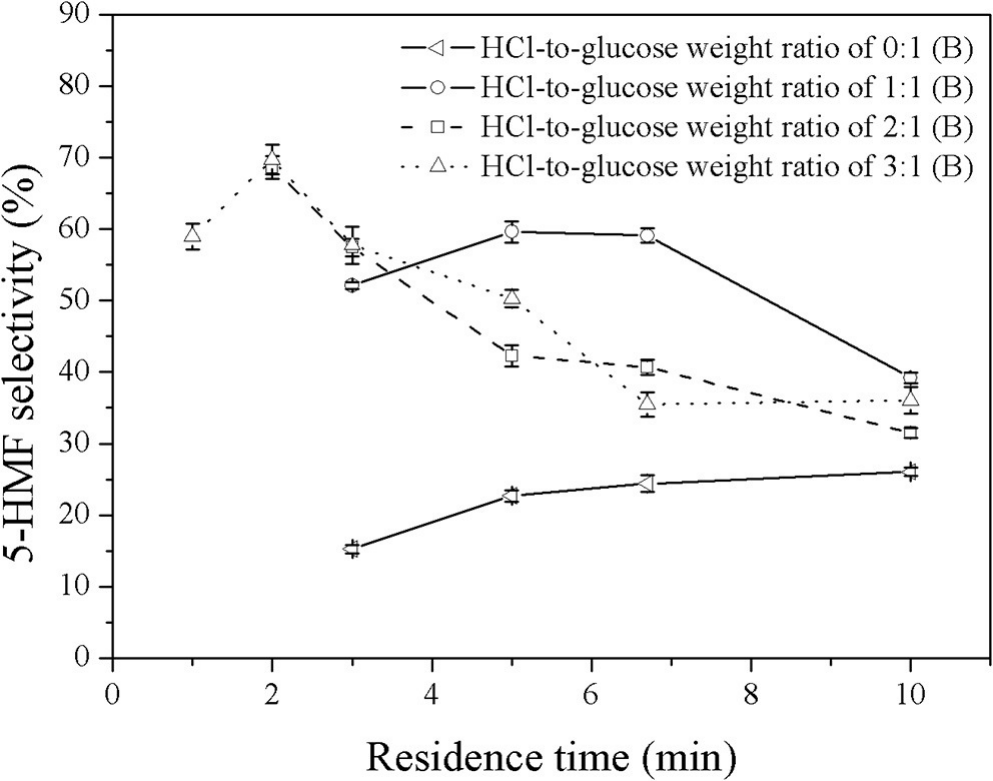
Effect of catalyst concentration on the selectivity of 5-HMF produced in a biphasic system under various residence times.

In this work, MIBK was applied as solvent to selectively extract 5-HMF from the aqueous phase, preventing further reactions of 5-HMF into undesired products. In order to optimize the operating conditions, several operating variables were investigated as follows; volumetric ratios of organic to aqueous phase (0.2:1 to 5:1), weight ratios of HCl to glucose (1:1 to 3:1), and residence times (2–10 min). The first variable was the weight ratio of HCl to glucose. Figures 5, 6 show the effect of catalyst concentration on the yield and selectivity of 5-HMF at different residence times and weight ratios of HCl to glucose, where the volumetric ratio of organic to aqueous phase of 1:1 was kept constant. As expected, a similar behavior was observed as compared to that of the monophasic system. An increase in catalyst concentration promoted both 5-HMF yield and selectivity, indicating that the direct dehydration of glucose into 5-HMF was possible with high concentration of Brønsted acid (HCl). For high HCl-to-glucose weight ratios (high catalyst concentration), a shorter residence time was required to avoid further conversion of 5-HMF to other by-products since large amount of 5-HMF was rapidly produced in the system. This result was in good agreement with the report of Chheda et al. (2007). An extremely low yield of 5-HMF was obtained in the biphasic catalyst-free system proving that the catalyst is required to obtain reasonably high yield of 5-HMF with relatively short residence time. As shown in Figure 5, a significant increase in the yield of 5-HMF produced in the biphasic system was observed when compared to that of the monophasic system (see Figure 3). This was owing to the suppression of further conversion of 5-HMF into undesired products as the 5-HMF was efficiently extracted into the organic phase. This also led to the shifting of the equilibrium reaction of glucose toward the formation of 5-HMF through the direct dehydration of glucose. This result verified that the extraction of 5-HMF from the aqueous phase into the organic phase is also one of the major factors to control the yield of 5-HMF. Moreover, a significant increase in the yield and selectivity of 5-HMF from the biphasic system was observed as the concentration of catalyst increased. This was because an increase in the catalyst concentration led to a larger number of acid sites, accelerating the direct dehydration of glucose into 5-HMF. The highest yield of 5-HMF of 52.6% was achieved at the weight ratio of HCl to glucose of 3:1 and the residence time of 3 min (see Figure 5).

**Figure 7:**
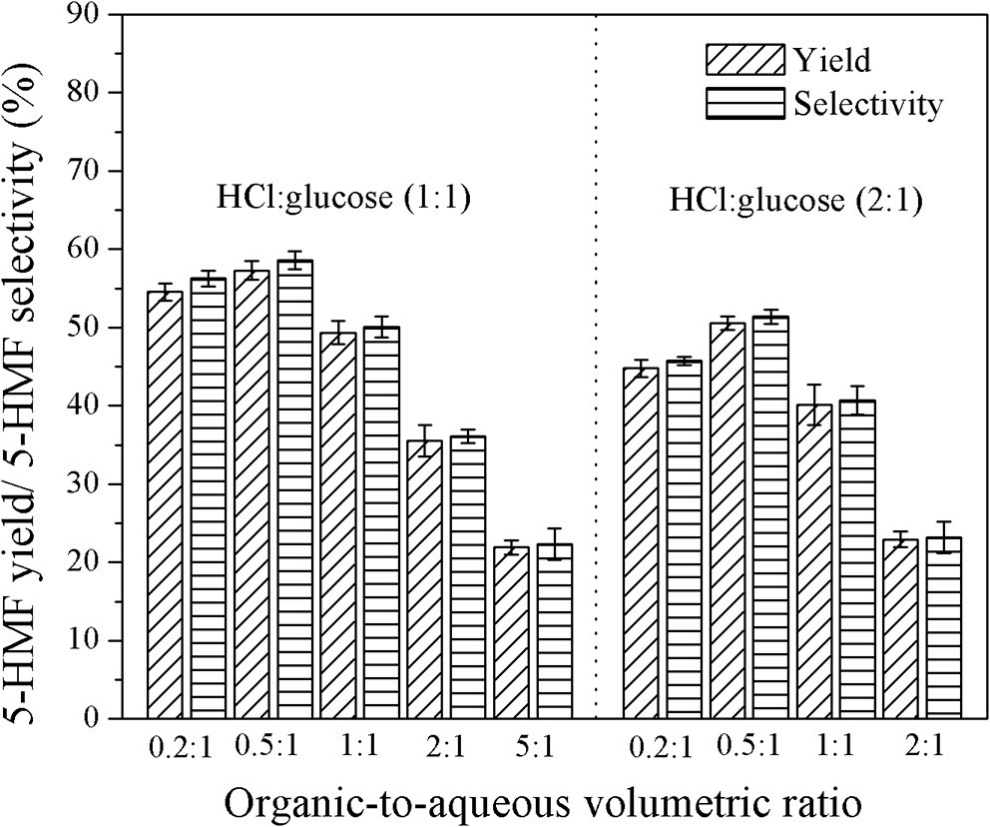
Effect of organic-to-aqueous volumetric ratio on the yield and selectivity of 5-HMF produced in a biphasic system for the residence time of 6.7 min.

**Figure 8:**
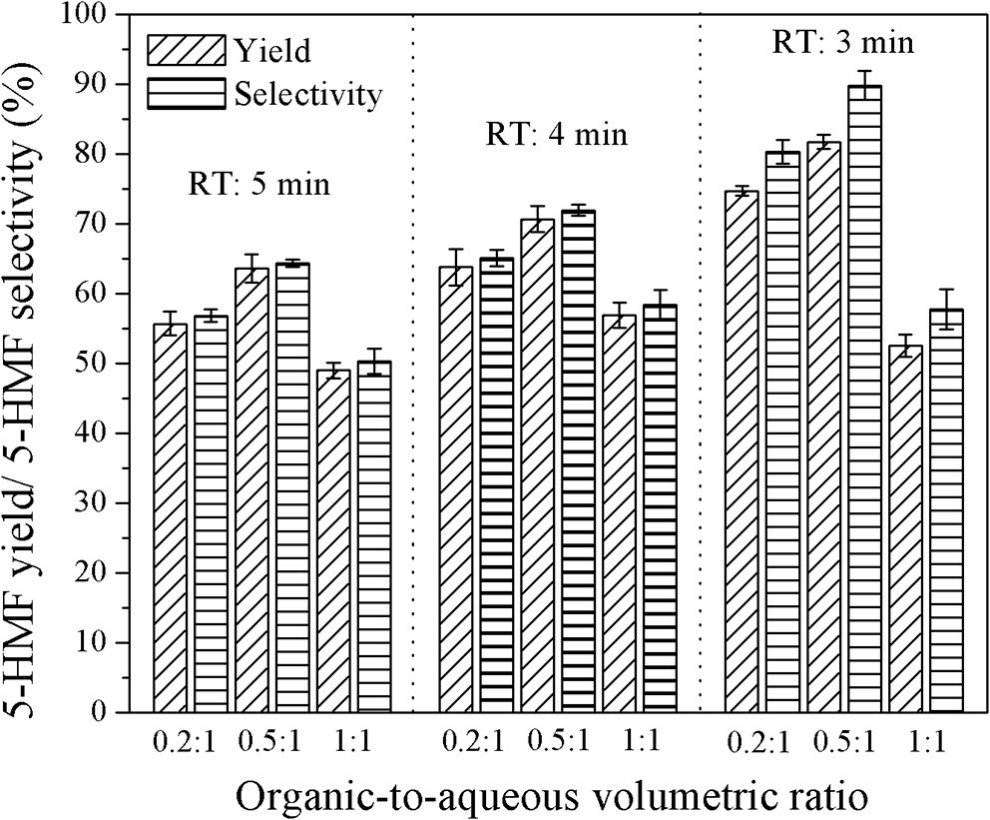
Effect of organic-to-aqueous volumetric ratio on the yield and selectivity of 5-HMF in a biphasic system for the catalyst ratio of 3:1.

The effect of organic-to-aqueous volumetric ratio on the yield and selectivity of 5-HMF was investigated in the range of 0.2:1 to 5:1 under various catalyst concentrations and residence times, whereas the reaction temperature was held constant at 180°C. Results are shown in Figure 7. At low organic-to-aqueous volumetric ratios (0.2:1 and 0.5:1) and weight ratio of HCl to glucose of 1:1 (see Figure 6), an increase of the organic-to-aqueous volumetric ratio from 0.2:1 to 0.5:1 slightly promoted the yield and selectivity of 5-HMF from 54.6 to 57.3% and from 56.3 to 58.6%, respectively. This was because the larger amount of MIBK in the system transferred 5-HMF from aqueous phase into the organic phase more effectively, preventing the rehydration of 5-HMF into undesired products (Leshkov and Dumesic, 2009; Dalessandro and Pliego, 2018). Higher extraction efficiency was strongly related to the mass transfer rate of 5-HMF into the organic phase (Zhou et al., 2018). However, the negative effect on the yield and selectivity is observed (see Figure 7) when the organic-to-aqueous volumetric ratio was higher than 0.5:1. With increasing the amount of MIBK in the system, the possibility that MIBK phase was contaminated with water also increased. As more water was able to dissolve into the MIBK phase, the hydration reaction between 5-HMF and water in the MIBK phase took place to a greater extent leading to the decline in both yield and selectivity of 5-HMF. A similar trend was found when the weight ratios of HCl to glucose of 2:1 (see Figure 7) and 3:1 (see Figure 8) were applied. Our results were in line with the report of Shimanouchi et al. (2016) who synthesized 5-HMF from fructose in a microtube reactor and found that the yield of 5-HMF decreased the volumetric ratio of organic to aqueous exceeded 1.25:1. Note that the yield and selectivity of 5-HMF decreased with increasing HCl weight ratio. Again, this was due further conversion of 5-HMF to undesired products under the prolonged reaction time (6.7 min).

**Figure 9:**
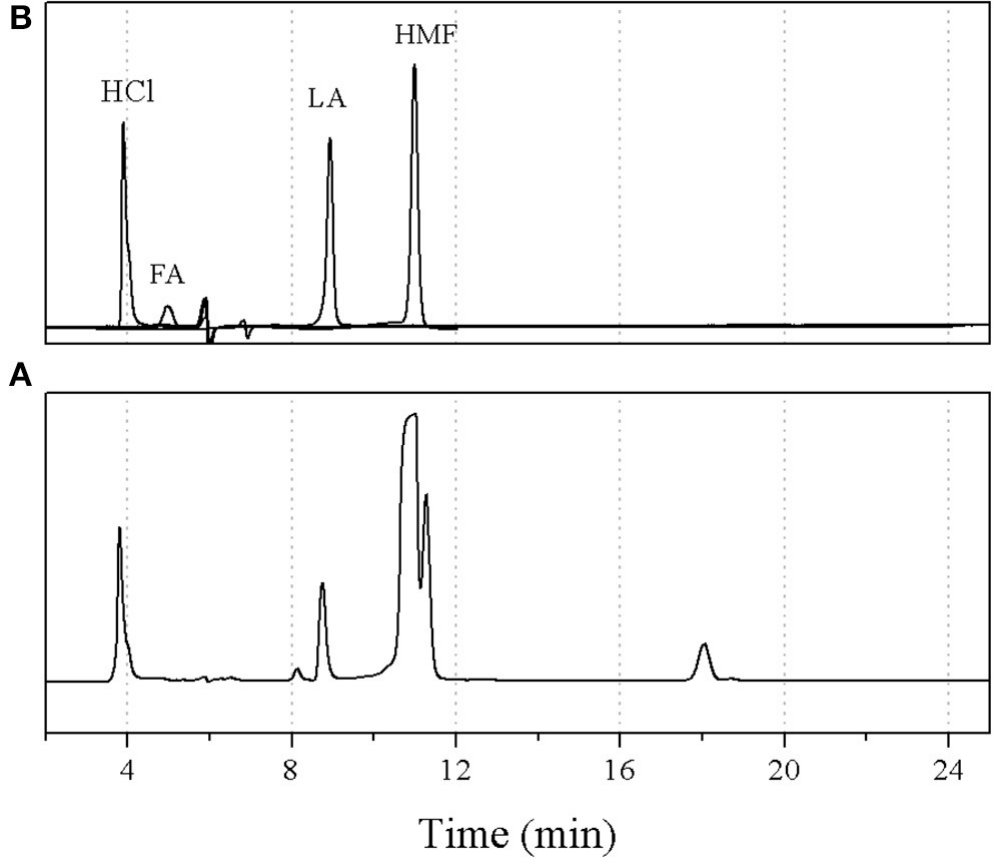
HPLC chromatograms of **(A)** sample at optimal conditions and **(B)** standards; hydrochloric acid (HCl), formic acid (FA), levulinic acid (LA), and hydroxymethylfurfural (HMF).

Figure 8 shows the effect of the organic-to-aqueous volumetric ratio on the yield and selectivity of 5-HMF at various residence times. The highest 5-HMF yield and selectivity of 81.7 and 89.8%, respectively, were achieved when using the organic-to-aqueous volumetric ratio of 0.5:1, reaction temperature of 180°C, and weight ratio of HCl to glucose ratio of 3:1. This was the highest yield reported so far for the synthesis of 5-HMF from glucose, demonstrating the enhancement of 5-HMF synthesis thought our method. The other products were formic acid and levulinic acid, confirmed by comparing with the analytical standards as shown in Figure 9.

**Figure 10:**
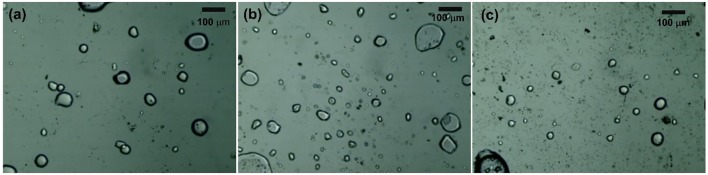
Flow pattern of our reacting mixture at the outlet of micromixer **(a)** O/A of 1:0.5, **(b)** O/A of 1:1, and **(c)** O/A of 1:1.5.

Furthermore, we compared these findings to the related results from other systems reported in the literature. The superior performance of our synthesis was evident. Only a small amount of MIBK was sufficient to effectively improve the yield and selectivity of 5-HMF. This was quite different to various reports the literature indicating relatively larger amount of MIBK in order to optimize the yield (the MIBK-to-aqueous volumetric is normally between 2:1 and 4:1). For example, in the work of Lueckgen et al. (2018) who applied a microtube reactor to synthesize 5-HMF from fructose, the volumetric ratio of MIBK-to-aqueous phase of 4:1 was necessary to obtain the maximum yield of about 80%. Another example was reported in the work of Delbecq et al. (2017) who studied the conversion of glucose to 5-HMF in a microwave-assisted batch reactor. The yield of 5-HMF of 45% was obtained with the MIBK-to-aqueous volumetric ratio of 3:1. Other related examples can be found elsewhere (Moreno-Recio et al., 2016; Jiang et al., 2018). The superior performance of our process was associated to the efficient extraction of 5-HMF from the aqueous phase due to the dispersed flow of the organic-aqueous mixture in our system. This was confirmed by observing the cloudy liquid product at the outlet of the reactor. This behavior was also investigated through a particular set of experiments to observe the flow pattern at the outlet of the T-micromixer. A stream of glucose solution mixed with green dye (to improve the visibility) and a stream of pure MIBK were used as feed streams. The experiments were carried out under ambient temperature, while the flow rate of aqueous stream and the flow rate of organic stream were the same as those of the reaction test conditions with the residence time of 3 min, and aqueous-to-organic phase of 0.5:1; 1:1; and 1:1.5. As shown in Figure 10, the dispersed flow was observed due to the presence of small droplets surrounded by the continuous phase, suggesting that mixing of aqueous and organic streams in our system was efficient.

**Figure 11:**
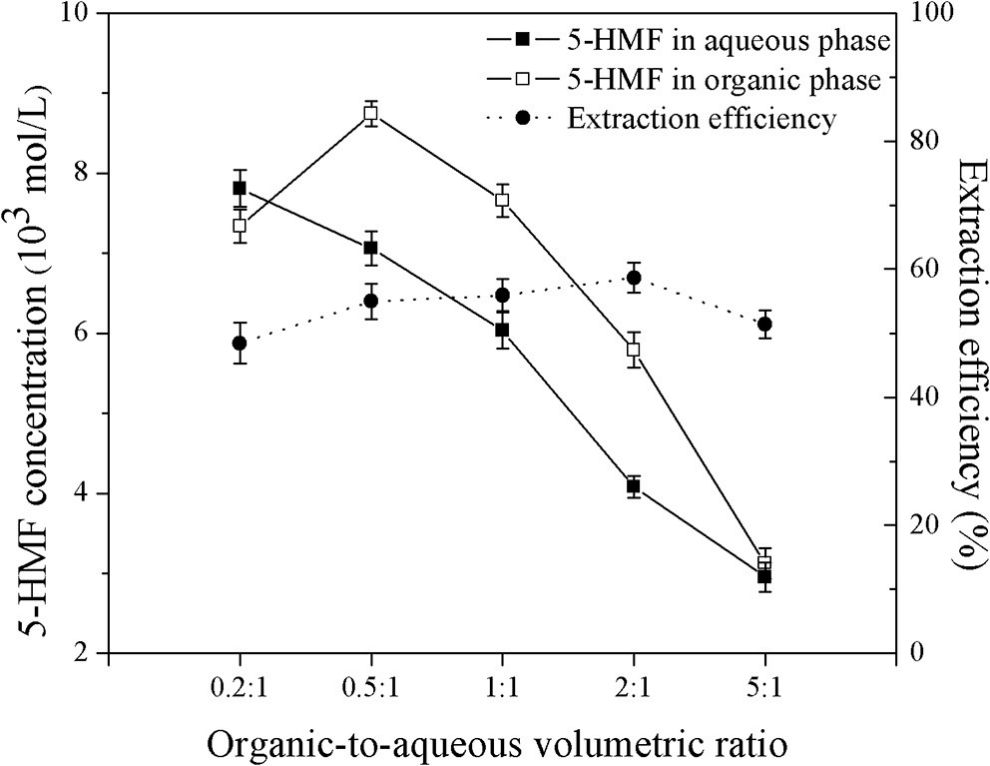
Effect of organic-to-aqueous volumetric ratio on the extraction efficiency.

The effect of organic-to-aqueous volumetric ratio on the yield and selectivity of 5-HMF can be interpreted by the extraction efficiency as shown in Figure 11. The results obtained at the residence time of 6.7 min, weight ratio of HCl to glucose of 1:1, and reaction temperature of 180°C showed that, the extraction efficiency was approximately the same throughout the range of organic-to-aqueous volumetric ratio investigated. Again, due to the increased possibility of water to contaminate in the organic phase, the hydration reaction between 5-HMF and water also took place in the organic phase. Consequently, the concentration of 5-HMF in both phases decreased rapidly with increasing the organic-to-aqueous volumetric ratio. These results were in line with the yield and selectivity results shown in Figure 7. Similar results were also reported in the work of Lueckgen et al. (2018).

### Product Purification

**Figure 12:**
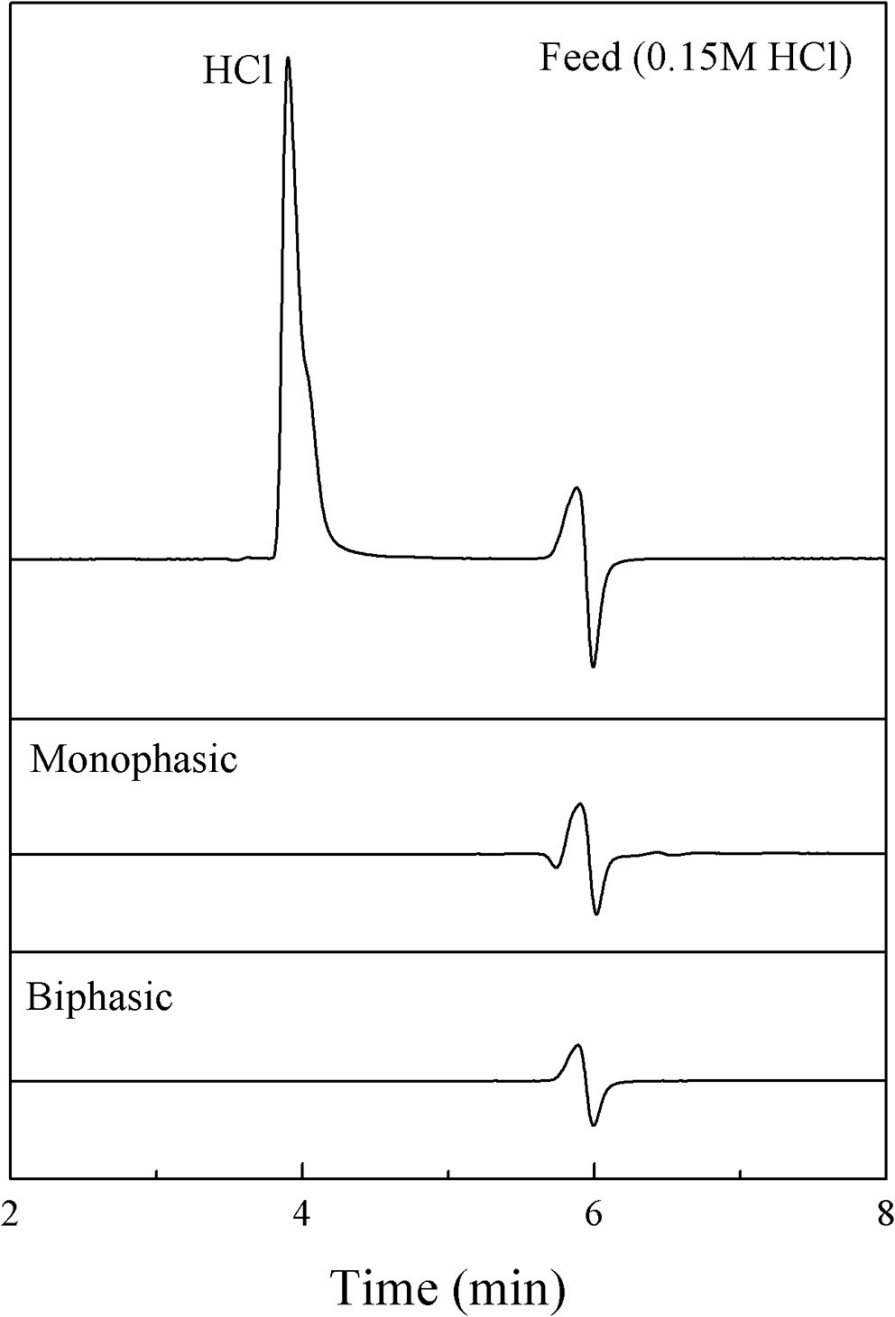
HPLC chromatograms of feed and product from the aqueous phase in both monophasic and biphasic systems.

Since the product obtained from the synthesis was contaminated with catalyst, the purification of 5-HMF was required. Note that, HCl is not soluble in an organic phase. Hence, we focused on a method for the removal of HCl (catalyst) from the aqueous product. A continuous process was developed by using a ion-exchange resin (Amberlyst A21) in order to selectively adsorb HCl. The amount of 1.5 g of resin was placed in a mini-packed bed (ID: 1/4”, 5.7 mL), which was used as a separator. A syringe pump was used to introduce the aqueous product through the bed. To verify our method, 0.15 M HCl in the aqueous phase was used as a model solution. Mini-packed bed reactor packed with ion-exchange resin (Amberlyst A21) with the amount of 1.5 g was used as a separator for both monophasic and biphasic systems. For the monophasic system, Only a stream of hydrochloric solution was fed through the adsorbent. On the other hand, a stream of MIBK was also mixed the stream of HCl solution before passing through the bed. The experiments were carried out at an ambient temperature at various residence times (3–20 min). The amount of HCl (in the aqueous phase) at the outlet of the separator was analyzed by HPLC using the same method as for the analysis of 5-HMF. Figure 12 shows that the trace of HCl was not detected from the aqueous phase for all residence times studied (3–20 min) for both cases (monophasic and biphasic system with a volumetric ratio of 0.5:1). These results suggested that the separation time of 3 min was sufficient for the removal of HCl from aqueous product. Hence, the product was effectively free of HCl.

**Figure 13:**
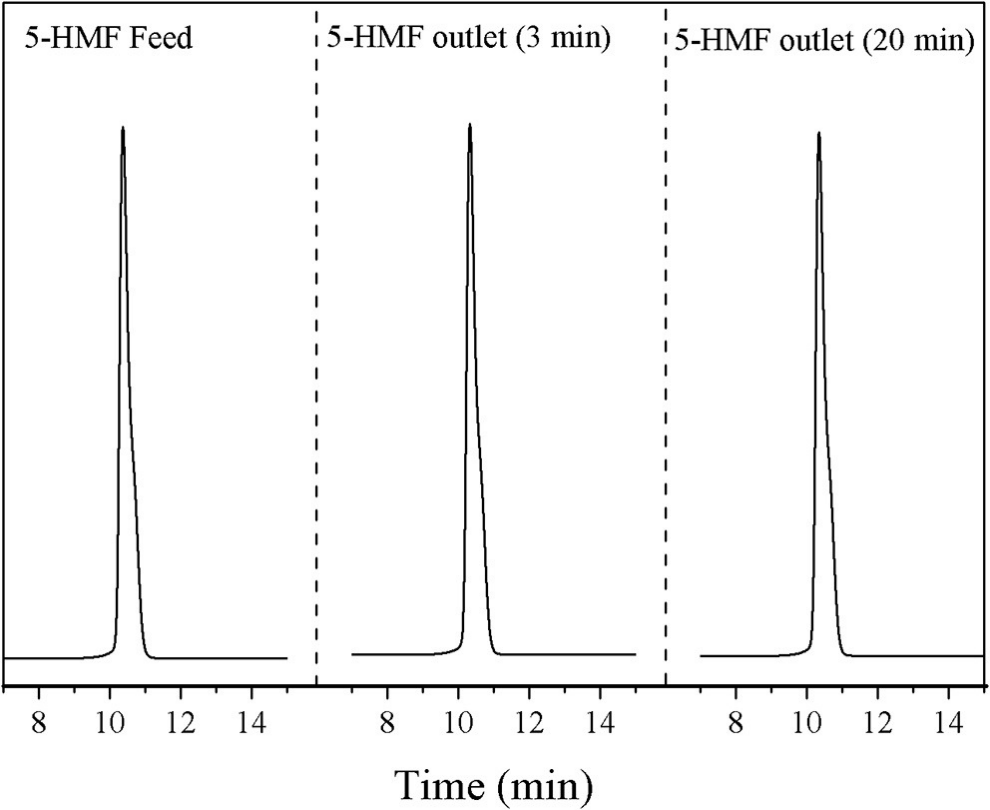
HPLC chromatograms of 5-HMF feed and 5-HMF outlet.

Moreover, similar experiments were performed to study the loss of 5-HMF during the separation of HCl. In these experiments, 5-HMF concentration at the optimal condition (81.7% of yield) was used as a feed solution. No significant losses of 5-HMF were observed at the stream outlet for the separation time of 3–20 min (see Figure 13). This result suggested that the *in-situ* processes of 5-HMF production and acidic purification is possible. However, further studies for optimizing the separation conditions should be performed. For the removal of solvent from 5-HMF, an evaporator can be used.

### Process Performance Comparison

To compare the process performance, 5-HMF yield obtained from our process was compared with those obtained from the related literature that employed different reactor types and operating conditions as summarized in Table 1. Apparently, our microreactor system provided an impressive yield of 5-HMF of 81.7% while the residence time was considerably shorter than that of other systems. Our system also offered other advantages such as the use of low catalyst concentration and small amount of organic solvent. This implied a smaller reactor volume required as well as a much reduced cost of solvent separation. Besides, when comparing the different types of reactors, the 5-HMF synthesis in a microreactor was apparently more effective than those performed in a batch reactor. This was due to the effective transfer of 5-HMF through the interface between organic phase and aqueous phase, while the batch reactor was affected by the dilution and small interfacial areas for the transfer of 5-HMF. For the cases of microreactor, the 5-HMF synthesis under a dispersed-flow condition (our method) provided superior performance compared to those performed under a slug-flow condition (Muranaka et al., 2017; Guo et al., 2019). From the results, it was evident that our process would be more economical due to the lower requirement of operating conditions. In addition, the production capacity of the microreactor system would be easily adjusted via the numbering-up method.

**Table 1:** Comparison of 5-HMF synthesis from glucose with literature.

**Reactor**	**Catalyst**	**Organic solvent**	**Promoter**	**Ratio^c^**	**T^d^ (^°^C)**	**RT^e^ (min)**	**Yield (%)**	**References**
Batch reactor	0.2 M HCl	γ-valerolactone	NaCl	4:1	140	60	62.4	Li et al., 2017
Batch reactor	1 wt% Sn- zeolite/HCl	THF	NaCl	3:1	190	70	53.0	Yang et al., 2015
Batch reactor	4 wt% SnO_2_-Al_2_O_3_	DMSO	–	4:1	150	60	27.5	Marianou et al., 2018
Micro reactor^a^	0.4 M phosphoric acid	2-sec-butyl phenol	Sodium phosphate dihydrate	3:1	180	47	75.7	Muranaka et al., 2017
Micro reactor^a^	AlCl_3_/HCl	MIBK	NaCl	4:1	160	16	66.2	Guo et al., 2019
Micro reactor^b^	0.15 M HCl	MIBK	–	0.5:1	180	3	81.7	This work

a *Synthesized over slug-flow regime*.

b *Synthesized over dispersed-flow regime*.

c *Organic-to-aqueous volumetric ratio*.

d *Reaction temperature*.

e *Residence time*.

## Conclusion

The synthesis of 5-HMF from glucose using HCl as catalyst was enhanced through the use of microreactor technology performed under the dispersed-flow condition. The biphasic system was found to be an effective means to suppress undesired reaction of 5-HMF and improved the yield and selectivity of 5-HMF. The main effect of operating conditions on the yield and selectivity including reaction temperature, catalyst concentration, organic-to-aqueous volumetric ratio, and residence time was studied. According to the ranges of operating conditions studied in this work, the 5-HMF yield of 81.7% was achieved at the residence time of 3 min, organic-to-aqueous volumetric ratio of 0.5:1, reaction temperature of 180°C, and weight ratio of HCl-glucose of 1:1. Compared to the literature data, this system was considered more efficient in terms of contact time, catalyst concentration, and the amount of solvent required. The removal of HCl from the aqueous phase was achieved using a mini-packed bed reactor filled with ion-exchange resin.

## Data Availability Statement

The datasets generated for this study are available on request to the corresponding author.

## Author Contributions

TT, NA, AK, and AJ designed and interpreted the experiments and wrote the manuscript. TT and NA set up and performed the experiments under supervision of AK and AJ.

### Conflict of Interest

The authors declare that the research was conducted in the absence of any commercial or financial relationships that could be construed as a potential conflict of interest.
